# HPCAL1 promotes glioblastoma proliferation via activation of Wnt/β‐catenin signalling pathway

**DOI:** 10.1111/jcmm.14083

**Published:** 2019-03-06

**Authors:** Dongming Zhang, Xidong Liu, Xuebin Xu, Jianmeng Xu, Zhongjun Yi, Baochang Shan, Bing Liu

**Affiliations:** ^1^ Department of Neurosurgery Dongying People's Hospital Dongying Shandong China; ^2^ Department of Oncology Dongying People's Hospital Dongying Shandong China; ^3^ Department of Neurosurgery Dongying District People's Hospital Dongying Shandong China; ^4^ Department of Neurology Dongying District People's Hospital Dongying Shandong China; ^5^ Department of Neurosurgery The Affiliated Hospital of Weifang Medical University Weifang Shandong China

**Keywords:** calcium ion, glioblastoma, hippocalcin like‐1 protein, Wnt/β‐catenin

## Abstract

Glioblastoma (GBM) is the most prevalent primary malignancy of the central nervous system with obvious aggressiveness, and is associated with poor clinical outcome. Studies have indicated that calcium ion (Ca^2+^) can positively regulate the initiation of malignancy with regard to GBM by modulating quiescence, proliferation, migration and maintenance. Hippocalcin like‐1 protein (HPCAL1) serves as a sensor of Ca^2+^. However, the understanding of HPCAL1 activity in GBM is limited. The present study revealed that the gene *HPCAL1* was up‐regulated by Ca^2+^ in the tissues and cells of GBM. Ectopic expression of *HPCAL1* promoted proliferation of cells. Exhaustion of HPCAL1 inhibited cell growth not only in vivo, but also in vitro. In addition, HPCAL1 enhanced the Wnt pathway by stimulating β‐catenin accumulation and nuclear translocation in GBM cells, while β‐catenin silencing significantly inhibited the proliferation and growth of the GBM cells. Our results showed that Ser9 phosphorylation of GSK3β was significantly decreased after *HPCAL1* knockdown in GBM cells, and knockdown of the gene *GSK3*β in GBM cells enhanced cell proliferation and promoted transcription of the genes *CCND1* and *c‐Myc*. Furthermore, the phosphorylation of ERK was decreased in the cells with *HPCAL1* knockdown, while it was promoted via overexpression of *HPCAL1*. The suppression or depletion of the gene *ERK* decreased proliferation triggered by overexpression of *HPCAL1* and impaired transcription of the genes *c‐Myc* and CCND1. These studies elucidate the tumour‐promoting activity of HPCAL1. They also offer an innovative therapeutic strategy focusing on the HPCAL1‐Wnt/β‐catenin axis to regulate proliferation and development of GBM.

## 1 INTRODUCTION

Glioblastoma (GBM) is the most prevalent primary cancer with obvious aggressiveness in human brain with poor clinical outcome.^1^ Despite the developments in treatment approaches, therapy of GBM is still challenging.^2^ The 1‐year survival rate of GBM patients is 36.5%.^3^ Even with numerous clinical trials conducted with different agents, there has been no proven success in terms of efficacy.^4^ Furthermore, the understanding of the biology of glioma is limited. Calcium ion (Ca^2+^) has been regarded as a dominant secondary messenger in eukaryotic cells. Emerging reports indicate that Ca^2+^ could serve as an essential positive modulator, as it influences the progression of GBM via enhanced quiescence, proliferation, migration and maintenance of malignant cells.^5^ Considering treatment approaches that block structures relying on Ca^2+^, such as channels and pumps, can never cure GBM.^5^ However, various approaches relying on Ca^2+^ utilization by cells in order to conquer checkpoints can be exploited to reprogram malignant stem cells to a different destiny.

The visinin‐like protein (VILIP) superfamily, includes VILIP1, VILIP2, VILIP3 (also called HPCAL1), neurocalcin‐δ and hippocalcin.^6^, ^7^, ^8^ Biological activities of the proteins belonging to this family are atypical. It has been reported that VILIP1 suppresses the invasiveness and proliferation of squamous cell carcinoma cells via inhibiting the function of matrix metalloproteinase‐9 and RhoA. Furthermore, VILIP1 also inhibits cancer progression via down‐regulation of α5 and αV integrins.^9^ Some studies have proved that the expression of *HPCAL1* mainly occurred in the Purkinje cells of brain, and the protein HPCAL1 might participate in the regulation of neuron types.^6^ Studies have also indicated that reinforced expression of *HPCAL1* stimulated ERK2 and its expression.^10^ It has been recognized that HPCAL1 is an innovative inhibitor of liver cancer, which was down‐regulated in the hepatocellular carcinoma (HCC) tissues and cells. Suppressed *HPCAL1* expression worsened clinical outcome in patients with HCC.^11^ On the contrary, interaction between HPCAL1 and wild‐type paired‐like homeobox 2b (WT PHOX2B) influenced the outgrowth of neurites in human neuroblastoma cells with *PHOX2B* expression. Elimination of the interaction by *HPCAL1* knockdown with small hairpin RNA (shRNA) in the neuroblastoma cells with PHOX2B expression reduced the outgrowth of neurites. Furthermore, the transcriptional profile predicted suppressed differentiation of sympathetic neurons.^12^ The understanding of the influence of HPCAL1 on the development of GBM cells is limited.

The present study evaluated the effect of: (a) overexpression of *HPCAL1* in the tissues and cells obtained from patients with GBM, and (b) abnormally stimulated Wnt/β‐catenin axis in order to enhance cell growth. Stimulation of HPCAL1 was regulated via Ca^2+^ concentration within the cells, which enhances ERK stimulation and inhibits the enzyme glycogen synthase kinase 3 beta (GSK3β). The findings of the present study will elucidate the innovative effects of HPCAL1 on progression of GBM, and also offer a promising strategy to treat GBM.

## 2 MATERIALS AND METHODS

### 2.1 Clinical specimens

Specimens were obtained from 19 sporadic patients with GBM and 17 healthy counterparts matched by age in Dongying People's Hospital, Dongying, Shandong, China. Written informed consent was acquired prior to the therapy and surgery from every participant. Nineteen pairs of GBM and the surrounding non‐cancer tissues were sampled. Before using those samples in medical research, consent of the participants and approval of Ethics Committee of the Dongying People's Hospital were acquired. Furthermore, anonymity was offered. Every sample was subjected to pathological verification. Classification was carried out in conformity with WHO criteria.

### 2.2 Cell culture and transfection

The normal neuronal cell lines, such as HCN‐1A and HCN‐2, and also GBM cell lines, such as temozolomide (TMZ) sensitive cell lines (U‐87MG, A172), and TMZ resistant cell lines (U‐138MG, LN‐229, U‐118MG, LN‐18)^13^ were procured from American Type Culture Collection (Manassas, VA, USA). The cell lines were preserved at 37°C with 5% carbon dioxide. Dulbecco's modified Eagle medium (DMEM) consisting of Ham's F12 medium (1:1) (Invitrogen) was mixed with 10% foetal bovine serum (FBS) (HyClone, Logan, UT, USA).

Transfection of small interfering RNA (siRNA) and plasmid was carried out using Lipofectamine^®^ 2000 (Invitrogen).^14^ Plasmids expressing the gene *HPCAL1* were produced via insertion of HPCAL1 cDNA into pcDNA3.1 vector (Addgene, Cambridge, MA, USA). Preliminarily prepared siRNA (Santa Cruz Biotechnology, Santa Cruz, CA, USA) was utilized to knockdown the genes *HPCAL1*,* GSK3β, ERK* and *β‐catenin* in the U‐87MG, U‐118MG and A172 cell lines. Transduction was carried out using preliminarily prepared lentiviral shRNA vectors to steadily knockdown the gene *HPCAL1* in the cells U‐87MG, U‐118MG and A172. The shRNA vectors specific to *HPCAL1* (sh HP1, TRCN0000056363, and sh HP2, TRCN0000056364) were procured from Thermo Fisher Scientific Open Biosystems (Waltham, MA, USA). Puromycin was used for the selection of transfected cells.

### 2.3 Analysis of β‐catenin nuclear localization

Immunofluorescence was used to evaluate nuclear β‐catenin in the cells LN‐18 and A172.^14^ Nikon fluorescence microscope (type TS800) supplemented with a SPOT camera and imaging software was used for observation.

### 2.4 TOPflash reporter assay

Transfection assay was conducted with β‐catenin/T cell factor (TCF) reporter plasmid (TOPflash). The cells (1 × 10^4^) were transfected for 48 hours using 2 μg of pTOPflash, inactive pFOPflash (Millipore) and pSV40‐Renilla plasmid serving as an internal control (Promega). The function of luciferase was evaluated using a Dual‐Glo Luciferase Assay System (Promega).

### 2.5 RNA separation and real‐time quantitative polymerase chain reaction analysis

Ribonucleic acid was separated from tissues and cells using Trizol reagent (Invitrogen). Subsequently, 2 μg of RNA was supplemented with RQ1 DNase (Promega, Madison, WI, USA) to produce RNA without DNase. Complementary DNA (cDNA) was generated via avian myeloblastosis virus reverse transcriptase (AMV‐RT) (Promega) using 1 μg of RNA after treatment with DNase. RT‐qPCR was carried out. Fold alteration in the expression of gene was evaluated according to 2^−ΔΔCt^ method with GAPDH transcripts as reference.

### 2.6 Western blotting

Ten percent sodium dodecyl sulfate‐polyacrylamide gel electrophoresis (SDS‐PAGE) was carried out to isolate protein lysates, which were then moved to PVDF membrane (GE Healthcare, Buckinghamshire, Great Britain). Subsequently, immunoblotting and detection were carried out with Super Signaling (Pierce, Rockford, IL, USA). Antibodies used included: Ki‐67 (ab15580, Abcam), c‐Myc (MABE282, Millipore), HPCAL1 (ab154160, Abcam), tubulin (T9026, Sigma‐Aldrich), β‐catenin (#8480), p‐Erk (#4370), p‐GSK3β (#9323) (Cell Signaling Technology) and cyclin D1 (SC‐753, Santa Cruz Biotechnology). Antibodies were probed using anti‐rabbit or antimouse secondary antibody (Pierce) conjugated with horseradish peroxidase.

### 2.7 MTT assay

MTT assay (Promega) was used to evaluate the proliferation capability of cells. Plates with 96 wells (BD Biosciences, Bedfort, MA, USA) were used to plant the cells (1 × 103 cells/well) in DMEM. The growth of cells was examined for five consecutive days by supplementing 20 μl of MTT (5 mg/ml) (Sigma‐Aldrich) to every well 4 hours before incubation at 37°C. Subsequently, the reaction was ceased by adding 200 μl of dimethyl sulfoxide (DMSO) (Sigma‐Aldrich). The optical density (OD) value was measured at 570 nm.

### 2.8 In vivo tumour assay

Animal experiments were approved by the Institutional Animal Ethics Committee of Dongying People's Hospital. Female non‐obese diabetic combined immunodeficiency mice with impaired immune systems were used. The mice (*n* = 5) received subcutaneous injection of 1 × 10^6^ of parent A172 cells and HPCAL1 shRNA transfectant cells. The mice were examined for the initiation and progression of malignancy. The volume of cancer was calculated according to the formula: 4/3π (major axis/2 × minor axis/2). The cancer tissue was fixed in formalin before haematoxylin and eosin staining and marker analysis. The tissue was then cut into pieces and fixed in 10% formalin and then embedded with paraffin. Immunostaining was carried out with antibodies, such as c‐Myc, p‐ERK (Cell Signaling Technology, USA), β‐catenin (BD Biosciences) and Brdu (Abcam).

### 2.9 Statistical analysis

GraphPad Prism V software was used for statistical analysis. The data were regarded as significant at *P* < 0.05. The results are presented as means ± *SD*.

## 3 RESULTS

### 3.1 HPCAL1 was up‐regulated via Ca^2+^ in GBM in vivo and in vitro

**Figure 1:**
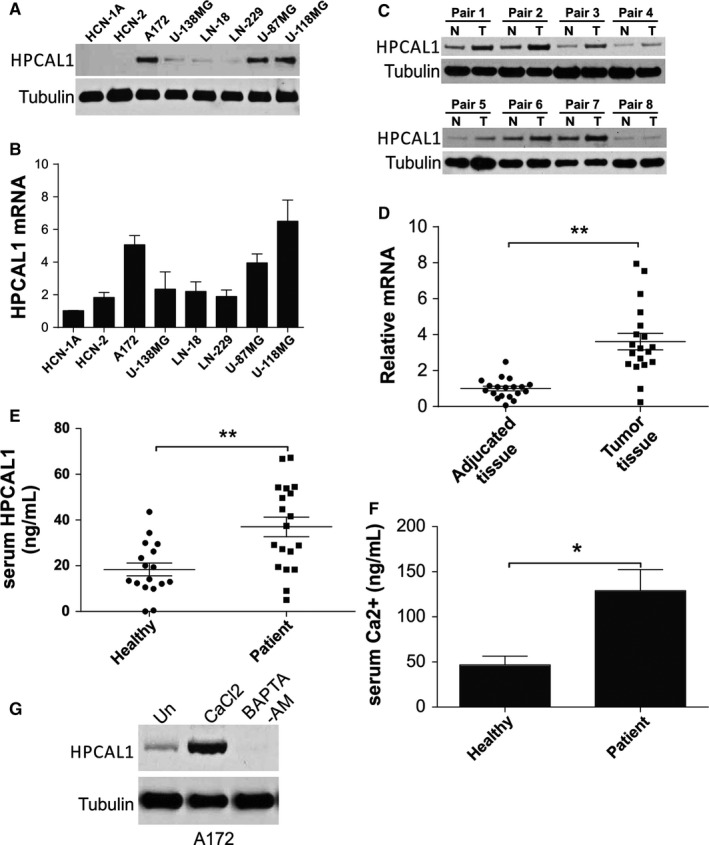
HPCAL1 up‐regulation in glioblastoma (GBM) was regulated via Ca^2+^ concentration. (A) HPCAL1 translation in GBM cells. (B) *HPCAL1* mRNA level in GBM cells. *N* = 3 for each cell lines. (C) Western blotting (WB) of HPCAL1 translation in GBM specimens and the surrounding counterparts of eight chosen pairs. (D) *HPCAL1* transcription in 19 pairs of GBM specimens and the surrounding counterparts. The ratio of mRNA level in tumour sample over the surrounding samples was presented. (E) HPCAL1 concentration in specimens of GBM patients (*N* = 19) and healthy participants (*N* = 17) was evaluated via ELISA. (F) Ca^2+^ concentration in specimens of GBM patients (*N* = 19) and healthy counterparts (*N* = 17). (G) A172 cells received 2 μmol/L calcium chloride or 3 μmol/L Ca^2+^ chelator (BAPTA‐AM) for 48 hours, and WB was used to evaluate the expression of *HPCAL1*. *P <* *0.05, **P <* *0.01. Western blots are representative pictures for two independent replicates

To investigate the influence of HPCAL1 on GBM, we firstly detected the protein and messenger RNA (mRNA) levels of HPCAL1 in various GBM cells. The results revealed that the expression of the gene *HPCAL1* was elevated in the majority of GBM cells in comparison with that of the normal cells, HCN‐2 and HCN‐1A (Figure 1A,B). However, the expression of HPCAL1 has no correlation with the TMZ response in GBM cells, as both TMZ‐resistant cells (U‐118MG) and TMZ‐sensitive cells (U‐87MG and A172) have higher HPCAL1 expression.^13^ The expression of the gene *HPCAL1* in 19 pairs of cancer tissues and the surrounding non‐cancer tissues was evaluated. Similar to the findings in cells, HPCAL1 transcription and translation were significantly promoted in the cancer tissues in comparison with those in non‐cancer tissues (Figure 1C,D). As the markers in circulation have been most frequently used for the diagnosis of cancer and diagnosis before surgery,^15^ the presence of HPCAL1 in blood was examined. The concentration of HPCAL1 was significantly enhanced in GBM patients in comparison with that of the healthy counterparts according to ELISA (Figure 1E). As HPCAL1 serves as a sensor of Ca^2+^, the present study verified if the up‐regulation of *HPCAL1* was associated with the concentration of Ca^2+^ in GBM patients. The concentration of Ca^2+^ was significantly promoted in patients with GBM, as expected (Figure 1E). Furthermore, activation via CaCl_2_ promoted the expression of *HPCAL1*, while supplementation of Ca^2+^ scavenger (BAPTA‐AM) exhibited opposite effects (Figure 1F). These findings suggest that *HPCAL1* expression was enhanced in GBM specimens, and it relied on Ca^2+^ concentration.

### 3.2 HPCAL1 enhanced GBM proliferation

**Figure 2:**
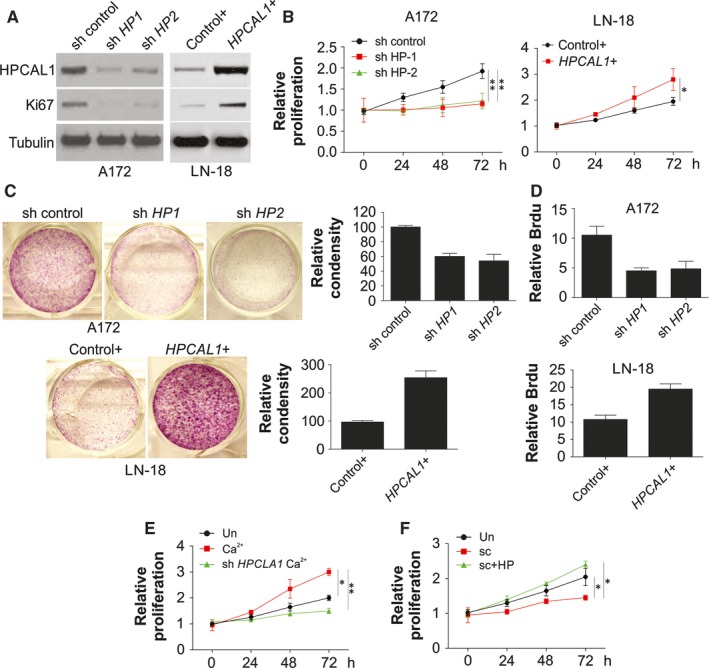
HPCAL1 regulates proliferation of GBM cells. (A) A172 cells were transfected steadily with two HPCAL1 shRNAs (sh HP1 and sh HP2) or control. LN‐18 cells were transfected with HPCAL plasmids or control. Western blotting was used to assess the expression of certain proteins. (B) A172 and LN‐18 cells were treated as in (A), MTT assay was adopted to evaluate the proliferation of cells at a specific time. The ratio of cell survival in different time points over beginning survival was presented, and the same thereafter. (C) Crystal violet staining method was employed to assess proliferation of LN‐18 as well as A172 cells mentioned in (B). (D) Brdu assay was used to evaluate the proliferation of LN‐18 and A172 cells mentioned in (B). (E) A172 cells after steady transfection with HPCAL1 shRNA (sh HP1, and the same thereafter) received 2 μmol/L calcium chloride. MTT assay was adopted to evaluate the proliferation of cells. (F) A172 cells after steady transfection with HPCAL1 plasmids received 3 μmol/L BAPTA‐AM. MTT assay was adopted to evaluate the proliferation of cells. *N* = 3 for (B)‐(F), *P <* *0.05, **P <* *0.01. Western blots are representative pictures for two independent replicates

To investigate the influence of HPCAL1 on biological activities of GBM cells, A172 cells, the high HPCAL1 expressed cells, and LN‐18 cells, the low HPCAL1 expressed cells, were chosen in the future study. The A172 cells with elevated *HPCAL1* expression were steadily exhausted via infection of two different shRNAs packing lentivirus (sh *HPCAL1*‐1 and sh *HPCAL1*‐2). The WB analysis revealed that the translation of HPCAL1 was significantly down‐regulated by both shRNAs (Figure 2A). The exhaustion of HPCAL1 noticeably suppressed the growth (Figure 2B,C) and proliferation (Figure 2A,D) of cells according to WB of Brdu and Ki67. As the effect of sh *HPCAL1*‐1 has better effect on HPCAL1 knockdown (Figure 2A), we used it in the following experiments. On the contrary, reinforced expression of the gene *HPCAL1* exhibited an opposite influence on proliferation in LN‐18 cells (Figure 2A‐D). Furthermore, HPCAL1 exhaustion in the A172 cells impaired the promotion of survival cells by Ca^2+^ overload (Figure 2E). The suppression effect of Ca^2+^ scavenger (BAPTA‐AM) was eliminated via overexpression of the gene *HPCAL1* (Figure 2F). These results indicate that HPCAL1 serves as a downstream effector of calcium and enhances the proliferation of GBM cells.

### 3.3 HPCAL1 enhanced stimulation of β‐catenin and Wnt target genes in GBM cells

**Figure 3:**
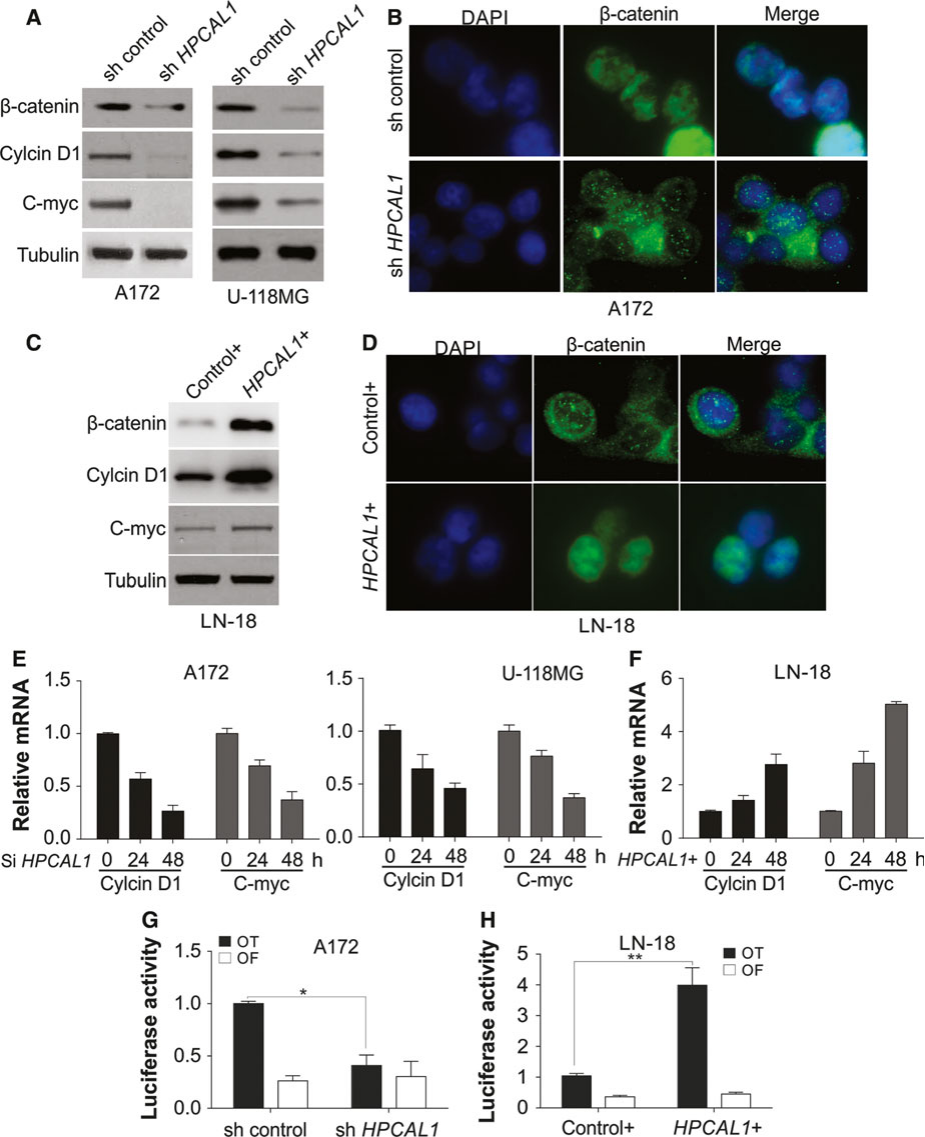
HPCAL1 enhances TCF‐4 function and β‐catenin nuclear translocation. (A) Western blotting (WB) of certain proteins from A172 and U‐118MG cells after steady transfection with HPCAL1 shRNA or control. (B) Nuclear β‐catenin translocation of A172 cells after steady transfection with HPCAL1 shRNA or control. (C) WB of certain proteins of LN‐18 cells after transfection with HPCAL1 plasmids or control. (D) Nuclear β‐catenin translocation of LN‐18 cells after steady transfection with HPCAL1 plasmids or control. (E) Modulation of c‐Myc and CCND1 transcription of A172 and U‐118MG cells after transfection with HPCAL1 siRNA at a specific interval. The ratio of mRNA in different time points over beginning was presented, and the same thereafter. (F) Modulation of c‐Myc and CCND1 transcription of LN‐18 cells after transfection with HPCAL1 plasmids at specific intervals. (G) A172 cells after steady transfection with HPCAL1 shRNA or the control underwent transfection with TCF‐4 reporter pTOPFlash (OT) or the control inactive reporter pFOPFlash (OF). Luciferase function after normalization was evaluated 24 hours after transfection. (H) LN‐18 was transfected with HPCAL1 plasmids or control together with OT or OF. Luciferase function after normalization was evaluated 24 hours after transfection. *N* = 3 for (E)‐(H), *P <* *0.05, **P <* *0.01. Western blots are representative pictures for two independent replicates

The present study examined the functions of HPCAL1 with regard to the enhancement of proliferation of GBM cells. A steady knockdown of *HPCAL1* in GBM cells, such as A172, U‐118G and U‐87MG cells, significantly suppressed the expression of Wnt target genes, such as *c‐Myc* and *CCND1* (Figure 3A; Figure S1A). Nuclear translocation of β‐catenin was also inhibited by depletion of HPCAL1 (Figure 3B; Figure S1B). On the contrary, the expression of *HPCAL1* in LN‐18 GBM cells, promoted the expression of the genes *c‐Myc* and *CCND1* (Figure 3C). Nuclear β‐catenin expression was also stimulated (Figure 3D; Figure S1C). The transcription modulation of *c‐Myc* and *CCND1* revealed that the exhaustion of HPCAL1 via siRNA inhibited their mRNA expression in A172, U‐118MG and U‐87MG cells (Figure 3E; Figure S1D). However, overexpression of *HPCAL1* promoted the expression of the two genes in LN‐18 cells (Figure 3F). Furthermore, *HPCAL1* knockdown in A172 cells suppressed TOPFlash reporter transactivation via WT β‐catenin (Figure 3G). On the contrary, overexpression of *HPCAL1* in LN‐18 cells promoted transactivation of WT β‐catenin TOPFlash reporter (Figure 3H). However, the influence of *HPCAL1* knockdown or overexpression on transactivation of TOPFlash reporter was eliminated via mutant β‐catenin, in which amino‐terminal phosphorylation sites (ΔN) necessary for its degeneration were not involved (Figure 3G,H). These results indicate that HPCAL1 enhanced the Wnt pathway by stimulating β‐catenin accumulation and nuclear translocation.

### 3.4 HPCAL1 modulated GBM progression by β‐catenin expression

**Figure 4:**
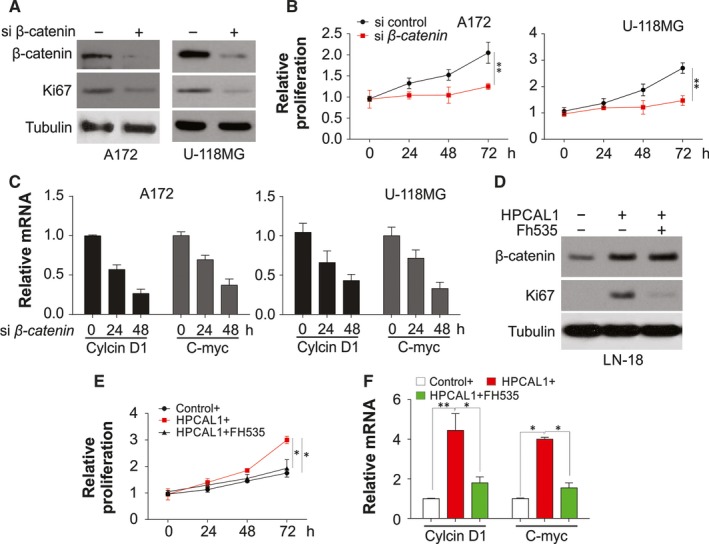
HPCAL1 enhances GBM cell proliferation through β‐catenin expression. (A) Western blot (WB) results of certain proteins from A172 and U‐118MG cells after transfection with β‐catenin siRNA or control. (B) Proliferation of A172 and U‐118MG cells after transfection with β‐catenin siRNA. (C) C‐Myc and CCND1 transcription of A172 and U‐118MG cells after transfection with β‐catenin siRNA or control. (D) LN‐18 cells after transfection with HPCAL1 plasmid or control supplemented with FH535 for 24 hours. WB was used to assess the expression of certain proteins. (E) MTT assay was adopted to evaluate the proliferation of LN‐18 cells mentioned in (D). (F) Modulation of c‐Myc and CCND1 transcription of LN‐18 cells mentioned in (D). *N* = 3 for (B), (C), (E), (F), *P <* *0.05, **P <* *0.01. Western blots are representative pictures for two independent replicates

As HPCAL1 can regulate β‐catenin pathway, the present study verified if the enhancement effect of HPCAL1 on growth relied on β‐catenin. Expression of β‐catenin was exhausted by siRNA in the cells with promoted *HPCAL1* expression. Furthermore, proliferation and growth of the cells were measured. The results revealed that β‐catenin silencing significantly inhibited the proliferation and growth of the cells A172, U‐118MG and U‐87MG (Figure 4A,B; Figure S2A,B). Exhaustion of β‐catenin inhibited transcription of the genes *c‐Myc* and *CCND1* indicating the suppression of Wnt/β‐catenin axis (Figure 4C; Figure S2C). The suppressor of β‐catenin, FH535 was adopted to verify if β‐catenin suppression decreased cell growth enhancement via overexpression of the gene *HPCAL1*. Supplementation of FH535 decreased growth and proliferation of cells via overexpression of *HPCAL1* (Figure 4D,E). Furthermore, the treatment with FH535 inhibited the transcription of the genes *c‐Myc* and *CCND1* via overexpression of the gene *HPCAL1* (Figure 4F). Similarly, depletion of β‐catenin by siRNA also compromised the effects of HPCAL1 overexpression on proliferation of LN‐18 cells (Figure S3A,B), and the transcription of *c‐Myc* and *CCND1* (Figure S3C). The results indicate that β‐catenin served as a downstream effector of HPCAL1 in the enhancement of proliferation and growth of GBM cells.

### 3.5 HPCAL1 stimulated β‐catenin expression via GSK3β phosphorylation

**Figure 5:**
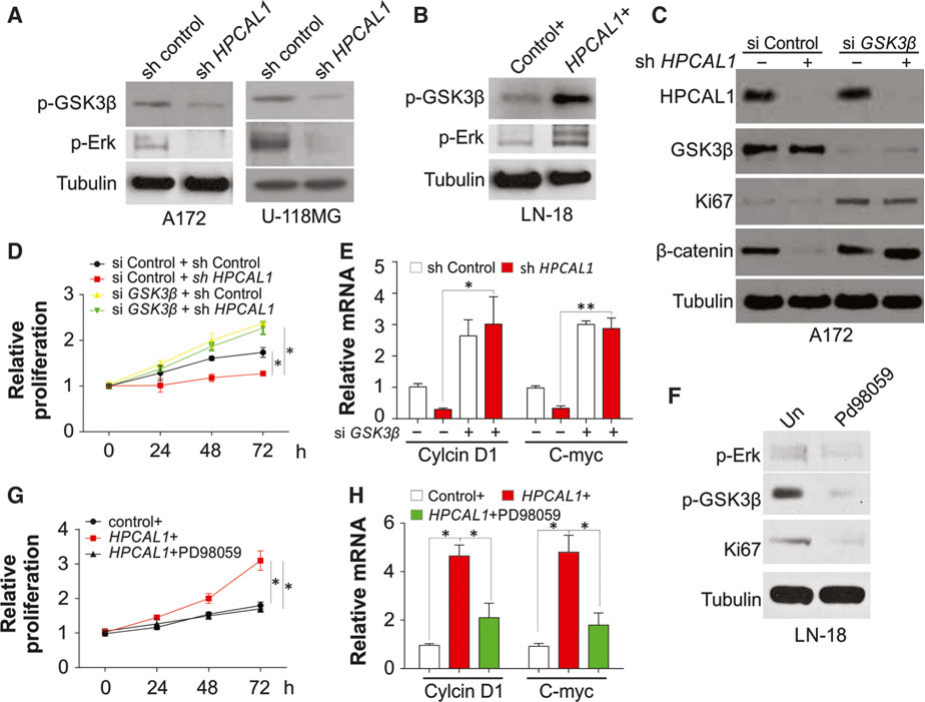
HPCAL1 stimulates β‐catenin via Erk/GSK3β in GBM cells. (A) Western blot (WB) results of certain proteins from A172 and U‐118MG cells after transfection with HPCAL1 shRNA or control. (B) WB results of certain proteins from LN‐18 cells after transfection with HPCAL1 plasmids or control. (C) Control of HPCAL1 shRNA stably expressed A172 cells were transfected with control or GSK3β siRNA, the expression of certain proteins was analysed by WB. (D) Proliferation of A172 cells treated as in (C). (E) C‐Myc and CCND1 transcription of A172 cells treated as in (C). (F) LN‐18 cells after transfection with HPCAL1 plasmid supplemented with 50 μmol/L Erk suppressor, PD98059, for 24 hours. WB was used to assess the expression certain proteins. (G) MTT assay was adopted to evaluate proliferation of LN‐18 cell mentioned in (F). (H) c‐Myc and CCND1 transcription of LN‐18 cell mentioned in (F). *N* = 3 for (D), (E), (G), (H), *P <* *0.05, **P <* *0.01. Western blots are representative pictures for two independent replicates

The present study also investigated the mechanisms of regulation of Wnt/β‐catenin axis via *HPCAL1* to enhance the progression of GBM. The concentration of β‐catenin was modulated via protein degeneration mediated by ubiquitin/proteasome after its phosphorylation via GSK3β,^16^ and its kinase function was determined via suppressed Ser9 phosphorylation.^17^ It was found that Ser9 phosphorylation of GSK3β was significantly decreased after *HPCAL1* knockdown in the cells A172, U‐118MG and U‐87MG (Figure 5A; Figure S1A), and promoted by overexpression of the gene *HPCAL1* in LN‐18 cells (Figure 5B). Knockdown of the gene *GSK3β* via siRNA in A172 cells enhanced proliferation (Figure 5C,D), activated the expression of β‐catenin (Figure 5C) and promoted transcription of the genes *CCND1* and *c‐Myc* (Figure 5E). Furthermore, absence of *GSK3β* also abolished the effects of HPCAL1 knockdown on cell proliferation, β‐catenin expression and transcription of *CCND1* and *c‐Myc* in A172 cells (Figure 5C‐E). HPCAL1 was reported to activate ERK kinase activtiy,^11^ and consequently enhanced Ser9 phosphorylation of GSK3β). We also observed that the phosphorylation of ERK decreased in the cells with *HPCAL1* knockdown (Figure 5A; Figure S1A), while it was promoted via overexpression of *HPCAL1* (Figure 5B). This indicated that HPCAL1 could deactivate GSK3β along with ERK. Similarly, ERK suppressor PD98059 inhibited Ser9 phosphorylation of GSK3β in LN‐18 cells, which overexpressed the gene *HPCAL1* (Figure 5F). The suppression of the gene *ERK* decreased proliferation triggered by overexpression of *HPCAL1* (Figure 5F,G) and impaired transcription of the genes *c‐Myc* and CCND1 (Figure 5H). Consistently, depletion of ERK by siRNA has the similar effects as PD98059 on LN‐18 cells, which was transfected with HPCAL1 plasmid (Figure S4A‐C). These findings suggest that HPCAL1 enhanced proliferation of GBM via stimulation of ERK and suppression of GSK3β, which further enhanced β‐catenin aggregation and nuclear translocation.

### 3.6 HPCAL1 suppressed cancer growth in nude mice

**Figure 6:**
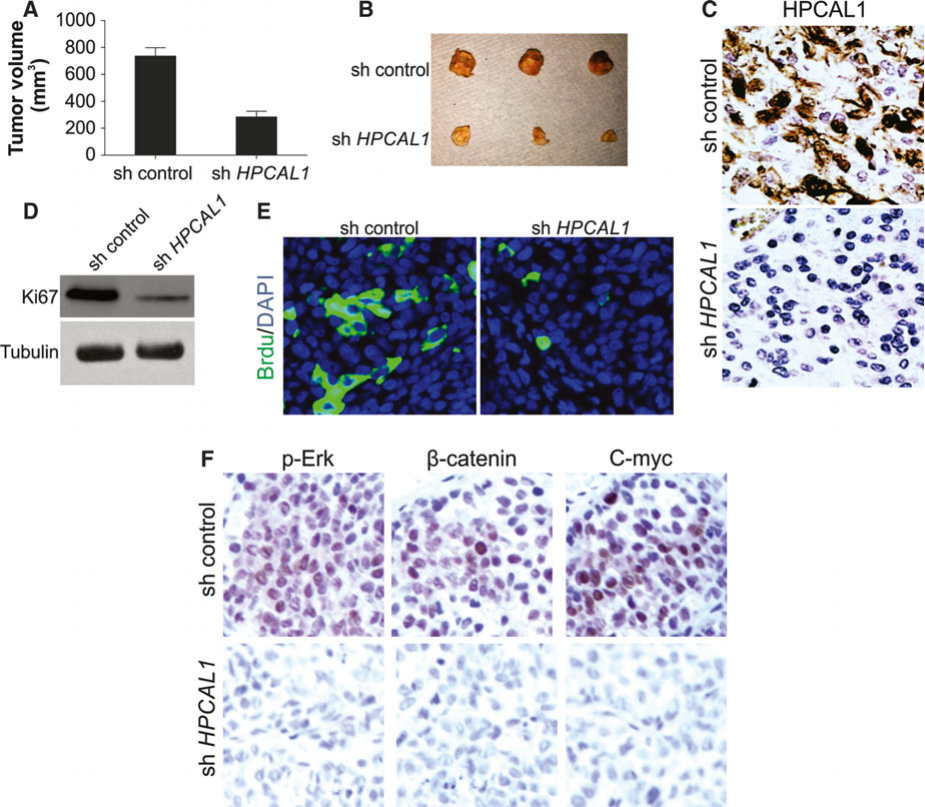
HPCAL1 enhances proliferation of GBM cell in vivo. (A) NOD‐SCID mice received subcutaneous injection of parent and HPCAL1 stably knockdown A172 cells to construct xenograft cancers. The volume of the malignancy was evaluated and recorded 3 weeks after the inoculation (*n* = 5/group). (B) Cancers at terminal stage in the experiment. (C) Representative immunochemistry staining of HPCAL1 in tumours from (B). (D) Western blotting (WB) of Ki67 in distinct groups of cancer from (B). (E) Representative immunostaining of Brdu in cancers from (B). (F) Immunochemistry staining analysis of p‐Erk, β‐catenin and c‐Myc in cancer specimens from (B). Western blots are representative pictures for two independent replicates

To determine the activity of HPCAL1 in vivo, mice were subcutaneously injected with A172 cells, containing HPCAL1, shRNA or specific empty vectors. The mice were executed 3 to 4 weeks after the injection. The cancer tissues were excised and examined. The average size of cancer was significantly smaller in *HPCAL1*‐knockdown group in comparison with that of the control group (Figure 6A,B). The immunochemical staining of HPCAL1 confirmed the knockdown effect of shRNA in vivo (Figure 6C). Simultaneously, proliferation of cells was retarded in *HPCAL1*‐knockdown cancers according to WB of Ki67 (Figure 6D) and immunostaining of Brdu (Figure 6E). Furthermore, the expression of the genes *c‐Myc* and *β‐catenin* was reduced in the *HPCAL1*‐knockdown cancer cells in comparison with that of the parent cancer cells (Figure 6F). This indicates that HPCAL1 enhanced proliferation of GBM not only in vivo, but also in vitro.

## 4 DISCUSSION

Glioblastoma is the most prevalent cancer with noticeable aggressiveness in brain. Relationship between cancer growth and proliferation indicates that various cancer stages preserve molecular features for a specific period of the development of autonomic system.^18^ Studies on regulation of proliferation and differentiation of malignant cells throw light on GBM and also an innovative therapeutic strategy. Emerging reports indicate that Ca^2+^ could play a crucial part in positive regulation of initiation of GBM by affecting the maintenance, migration, quiescence and proliferation of malignant cells.^5^ As calcium pathway has been recognized to regulate diverse cellular reactions, it can be presumed that the pathway influences the progression of malignancy.^19^ Up‐regulation of *HPCAL1* has been demonstrated in patients with GBM.^12^ In the present study, there was an up‐regulation of *HPCAL1* in GBM cells, which enhanced proliferation of GBM cells worsening clinical outcome in participants with GBM. Further, HPCAL1 stimulated phosphorylation of not only GSK3β, but also ERK, simultaneously with the stimulation of Wnt/β‐catenin axis, which influenced the proliferation of GBM cells.

Hippocalcin like‐1 protein (HPCAL1), a member of visinin‐like (VSNL) subfamily protein, is characterized by a strict pattern of expression in brain cells. It has been found in the granule cells, Purkinje cells^6^ and sympathetic ganglia during the developmental period. The VSNL family has been reported to exhibit specific activities with regard to signal transduction, membrane trafficking and differentiation among specific subgroups of neuronal cells. Each neuronal calcium sensor (NCS) protein was specific to several kinds of cells, receptors and pathways. The translocation capability of VSNL proteins from the cytoplasm to the subcellular membrane compartments, especially after an increase in the level of Ca^2+^ in cytoplasm, is due to the EF‐hand calcium‐binding motifs and the consensus N‐terminal myristoylation sequence.^6^, ^20^ Despite the dependence of HPCAL1 on Ca^2+^ in brain homogenates, few studies have demonstrated its influence on brain tumour. Several studies have also indicated that HPCAL1 enhanced neuroblastoma differentiation, which was impaired via interaction with PHOXB.^12^ However, the interaction between PHOXB and HPCAL1 does not rely on calcium. Consequently, the understanding of calcium sensor activity of HPCAL1 with regard to generation and progression of malignancies is limited. In our study, we found that HPCAL1 expression was positively associated with Ca^2+^ concentration within the cells. Escalated cellular Ca^2+^ usually results in mRNA level changes of Ca^2+^ binding proteins, such as regucalcin,^21^ VILIP‐1, Calbindin‐D28K.^22^ Besides, under disturbed Ca^2+^‐homeostasis condition, reduced expression of HPCAL1 lead to enhanced oxidative stress, and increased the apoptosis in neuron, which will result in Alzheimer's disease.^22^ Thus, increased expression of HPCAL1 may be protective for neuron against oxidative stress, and promotes neuron cell growth. Consistently, we also found that the increased Ca^2+^ level in GBM promote the expression of HPCAL1, and enhanced the GBM cell proliferation. Silence of HPCAL1 abolished the proliferation effect of Ca^2+^ in GBM cells (Figure 2E), but enhanced expression of HPCAL1 reversed the suppression effect of Ca^2+^ scavenger on GBM proliferation (Figure 2F). These results suggested that HPCAL1 should be the Ca^2+^ sensor in the GBM cell proliferation. However, the detail mechanism for HPCAL1 induction by Ca^2+^ level still remain unclear, and worth more efforts to be further investigated.

The up‐regulation of *HPCAL1* stimulated the Erk‐Wnt/β‐catenin axis, which led to the proliferation of GBM cells. Further, the present study highlighted the influence of HPCAL1 on enhancing the progression of GBM. Extracellular signal‐regulated protein kinases 1 and 2 are a part of mitogen‐stimulated protein kinase family, which regulates proliferation of cells. Several studies have suggested that stimulation of Ras‐Raf‐MEK‐ERK axis^23^, ^24^, ^25^ and Raf‐MEK‐ERK axis can result in glioma.^26^ In the present study, our results showed that the increased intracellular Ca^2+^ enhanced the expression of HPCAL1 followed by enhanced ERK activity. Consequently, the wnt/β‐catenin pathway was activated and promotes the proliferation ability of glioma cells. Furthermore, ERK and MEK were abnormally stimulated in gliomas and other malignancies,^27^ and could serve as an upstream event, and is necessary to deactivate GSK3β.^17^ Previous studies have demonstrated the interaction between ERK2 and HPCAL1.^11^ Accordingly, our results provided also suggested that the expression of HPCAL1 lead to the activation of ERK, which would deactivate GSK3β, and therefore constrain the activity of β‐catenin. However, the precise mechanism of interaction between ERK and HPCAL1 in GBM requires further exploration.

It has been demonstrated that the Wnt/β‐catenin axis participates in GBM^28^ The expression of β*‐*catenin was enhanced in several GBM cells, thus triggering Wnt target genes and promoting proliferation,^29^ invasion and migration.^30^ Findings of the present study suggest that HPCAL1 is an upstream effector of the Wnt/β‐catenin axis of GBM. This is implied by a close relationship between the promoted expression of HPCAL1, aggregation of c‐Myc, β‐catenin and CCND1 in GBM, and inhibition of Wnt via exhaustion of HPCAL1 in GBM cells. HPCAL1 can assist in preserving cytosolic β‐catenin, which binds to some proteins and enhances their turnover rate. Besides HPCAL1, other abnormalities of GBM can also stimulate Wnt, including overexpression of Wnt ligand^31^ and down‐regulation of antagonists of Wnt.^29^ The function of Wnt and malignant stemness was modulated via the micro‐environment, where agents, such as growth factor of liver cells generated via myofibroblasts, stimulated transcription relying on β‐catenin and promoted cancer stem cell clonogenicity.^32^ No mutation specific to β‐catenin in GBM has been reported. Promoter hypermethylation of Wnt pathway suppressors, such as NKD2 and sFRP2, has been frequently reported in numerous GBMs.^33^ Findings of the present study indicate that HPCAL1 stimulates the Wnt pathway by enhancing ERK and indirectly inactivating GSK3β, thus enhancing β‐catenin nuclear translocation. As β‐catenin is also involved in GBM metastasis,^30^ we believed that HPCAL1 might also have function to promote GBM cell invasion and migration. Therefore, further studies focusing on the expression of HPCAL1 that propels proliferation and migration of GBM are necessary.

The results of the present study proved the influence of HPCAL1 on the stimulation of Wnt/β‐catenin axis and proliferation of GBM. Future research on the functional effect of HPCAL1 can offer innovative strategies to identify pharmacological or biological targets of GBM.

## COMPETING INTERESTS

The authors confirm that there are no conflicts of interest.

## AUTHORS’ CONTRIBUTIONS

In this work, Dongming Zhang and Xidong Liu conceived the study and designed the experiments. Xuebin Xu, Jianmeng Xu and Zhongjun Yi contributed to the data collection, Baochang Shan and Bing Liu performed the data analysis and interpreted the results. Dongming Zhang and Xidong Liu wrote the manuscript; Dongming Zhang contributed to the critical revision of article. All authors read and approved the final manuscript.

## References

[1] Stupp R , Mason WP , van den Bent MJ , et al.; European Organisation for R, Treatment of Cancer Brain T, Radiotherapy G, National Cancer Institute of Canada Clinical Trials G . Radiotherapy plus concomitant and adjuvant temozolomide for glioblastoma. N Engl J Med. 2005;352:987‐996. 15758009 10.1056/NEJMoa043330

[2] Omuro A , DeAngelis LM . Glioblastoma and other malignant gliomas: a clinical review. JAMA. 2013;310:1842‐1850. 24193082 10.1001/jama.2013.280319

[3] Ostrom QT , Gittleman H , Liao P , et al. CBTRUS Statistical Report: primary brain and other central nervous system tumors diagnosed in the United States in 2010‐2014. Neuro Oncol. 2017;19:v1‐v88. 29117289 10.1093/neuonc/nox158 PMC5693142

[4] Reardon DA , Rich JN , Friedman HS , Bigner DD . Recent advances in the treatment of malignant astrocytoma. J Clin Oncol. 2006;24:1253‐1265. 16525180 10.1200/JCO.2005.04.5302

[5] Leclerc C , Haeich J , Aulestia FJ , et al. Calcium signaling orchestrates glioblastoma development: facts and conjunctures. Biochim Biophys Acta. 2016;1863:1447‐1459. 26826650 10.1016/j.bbamcr.2016.01.018

[6] Spilker C , Gundelfinger ED , Braunewell KH . Evidence for different functional properties of the neuronal calcium sensor proteins VILIP‐1 and VILIP‐3: from subcellular localization to cellular function. Biochim Biophys Acta. 2002;1600:118‐127. 12445467 10.1016/s1570-9639(02)00452-1

[7] Burgoyne RD , Weiss JL . The neuronal calcium sensor family of Ca^2+^‐binding proteins. Biochem J. 2001;353:3108‐12. PMC1221537 11115393

[8] Jheng FF , Wang L , Lee L , Chang LS . Functional contribution of Ca^2+^ and Mg^2+^ to the intermolecular interaction of visinin‐like proteins. Protein J. 2006;25:250‐256. 16703469 10.1007/s10930-006-9008-5

[9] Mahloogi H , Gonzalez‐Guerrico AM , Lopez De Cicco R , et al. Overexpression of the calcium sensor visinin‐like protein‐1 leads to a cAMP‐mediated decrease of in vivo and squamous cell carcinoma in vitro growth and invasiveness of squamous cell carcinoma cells. Cancer Res. 2003;63:4997‐5004. 12941826

[10] Spilker C , Braunewell KH . Calcium‐myristoyl switch, subcellular localization, and calcium‐dependent translocation of the neuronal calcium sensor protein VILIP‐3, and comparison with VILIP‐1 in hippocampal neurons. Mol Cell Neurosci. 2003;24:766‐778. 14664824 10.1016/s1044-7431(03)00242-2

[11] Zhang Y , Liu Y , Duan J , et al. Hippocalcin‐like 1 suppresses hepatocellular carcinoma progression by promoting p21(Waf/Cip1) stabilization by activating the ERK1/2‐MAPK pathway. Hepatology. 2016;63:880‐897. 26659654 10.1002/hep.28395

[12] Wang W , Zhong Q , Teng L , et al. Mutations that disrupt PHOXB interaction with the neuronal calcium sensor HPCAL1 impede cellular differentiation in neuroblastoma. Oncogene. 2014;33:3316‐3324. 23873030 10.1038/onc.2013.290 PMC4040330

[13] Lee SY . Temozolomide resistance in glioblastoma multiforme. Genes Dis. 2016;3:198‐210. 30258889 10.1016/j.gendis.2016.04.007 PMC6150109

[14] Chen X , Song X , Yue W , et al. Fibulin‐5 inhibits Wnt/beta‐catenin signaling in lung cancer. Oncotarget. 2015;6:15022‐15034. 25909283 10.18632/oncotarget.3609 PMC4558133

[15] Di Tommaso L , Destro A , Fabbris V , et al. Diagnostic accuracy of clathrin heavy chain staining in a marker panel for the diagnosis of small hepatocellular carcinoma. Hepatology. 2011;53:1549‐1557. 21520170 10.1002/hep.24218

[16] Clevers H . Wnt/beta‐catenin signaling in development and disease. Cell. 2006;127:469‐480. 17081971 10.1016/j.cell.2006.10.018

[17] Ding Q , Xia W , Liu JC , et al. Erk associates with and primes GSK‐3beta for its inactivation resulting in upregulation of beta‐catenin. Mol Cell. 2005;19:159‐170. 16039586 10.1016/j.molcel.2005.06.009

[18] Edsjo A , Holmquist L , Pahlman S . Neuroblastoma as an experimental model for neuronal differentiation and hypoxia‐induced tumor cell dedifferentiation. Semin Cancer Biol. 2007;17:248‐256. 16828305 10.1016/j.semcancer.2006.04.005

[19] Roderick HL , Cook SJ . Ca^2+^ signalling checkpoints in cancer: remodelling Ca^2+^ for cancer cell proliferation and survival. Nat Rev Cancer. 2008;8:361‐375. 18432251 10.1038/nrc2374

[20] O'Callaghan DW , Tepikin AV , Burgoyne RD . Dynamics and calcium sensitivity of the Ca^2+^/myristoyl switch protein hippocalcin in living cells. J Cell Biol. 2003;163:715‐721. 14638856 10.1083/jcb.200306042 PMC2173692

[21] Yamaguchi M , Ueoka S . Expression of calcium‐binding protein regucalcin mRNA in fetal rat liver is stimulated by calcium administration. Mol Cell Biochem. 1998;178:283‐287. 9546611 10.1023/a:1006803211864

[22] Braunewell KH . The visinin‐like proteins VILIP‐1 and VILIP‐3 in Alzheimer's disease‐old wine in new bottles. Front Mol Neurosci. 2012;5:20. 22375104 10.3389/fnmol.2012.00020 PMC3284765

[23] Hermanson M , Funa K , Hartman M , et al. Platelet‐derived growth factor and its receptors in human glioma tissue: expression of messenger RNA and protein suggests the presence of autocrine and paracrine loops. Cancer Res. 1992;52:3213‐3219. 1317261

[24] Dai C , Celestino JC , Okada Y , Louis DN , Fuller GN , Holland EC . PDGF autocrine stimulation dedifferentiates cultured astrocytes and induces oligodendrogliomas and oligoastrocytomas from neural progenitors and astrocytes in vivo. Genes Dev. 2001;15:1913‐1925. 11485986 10.1101/gad.903001 PMC312748

[25] Snuderl M , Fazlollahi L , Le LP , et al. Mosaic amplification of multiple receptor tyrosine kinase genes in glioblastoma. Cancer Cell. 2011;20:810‐817. 22137795 10.1016/j.ccr.2011.11.005

[26] Verhaak RG , Hoadley KA , Purdom E , et al.; Cancer Genome Atlas Research N . Integrated genomic analysis identifies clinically relevant subtypes of glioblastoma characterized by abnormalities in PDGFRA, IDH1, EGFR, and NF1. Cancer Cell. 2010;17:98‐110. 20129251 10.1016/j.ccr.2009.12.020 PMC2818769

[27] Roberts PJ , Der CJ . Targeting the Raf‐MEK‐ERK mitogen‐activated protein kinase cascade for the treatment of cancer. Oncogene. 2007;26:3291‐3310. 17496923 10.1038/sj.onc.1210422

[28] Kaur N , Chettiar S , Rathod S , et al. Wnt3a mediated activation of Wnt/beta‐catenin signaling promotes tumor progression in glioblastoma. Mol Cell Neurosci. 2013;54:44‐57. 23337036 10.1016/j.mcn.2013.01.001

[29] Rampazzo E , Persano L , Pistollato F , et al. Wnt activation promotes neuronal differentiation of glioblastoma. Cell Death Dis. 2013;4:e500. 23429286 10.1038/cddis.2013.32 PMC4098797

[30] Kim KH , Seol HJ , Kim EH , et al. Wnt/beta‐catenin signaling is a key downstream mediator of MET signaling in glioblastoma stem cells. Neuro Oncol. 2013;15:161‐171. 23258844 10.1093/neuonc/nos299 PMC3548587

[31] Ma Q , Yang Y , Feng D , et al. MAGI3 negatively regulates Wnt/beta‐catenin signaling and suppresses malignant phenotypes of glioma cells. Oncotarget. 2015;6:35851‐35865. 26452219 10.18632/oncotarget.5323 PMC4742146

[32] Vermeulen L , De Sousa EMF , van der Heijden M , et al. Wnt activity defines colon cancer stem cells and is regulated by the microenvironment. Nat Cell Biol. 2010;12:468‐476. 20418870 10.1038/ncb2048

[33] Gotze S , Wolter M , Reifenberger G , Muller O , Sievers S . Frequent promoter hypermethylation of Wnt pathway inhibitor genes in malignant astrocytic gliomas. Int J Cancer. 2010;126:2584‐2593. 19847810 10.1002/ijc.24981

