# Supplementary figures and images for: HPCAL1 promotes glioblastoma proliferation via activation of Wnt/β‐catenin signalling pathway
Source: J Cell Mol Med. 2019 Mar 6;23(5):3108–17. doi: 10.1111/jcmm.14083 (PMC6484330; doi:10.1111/jcmm.14083)

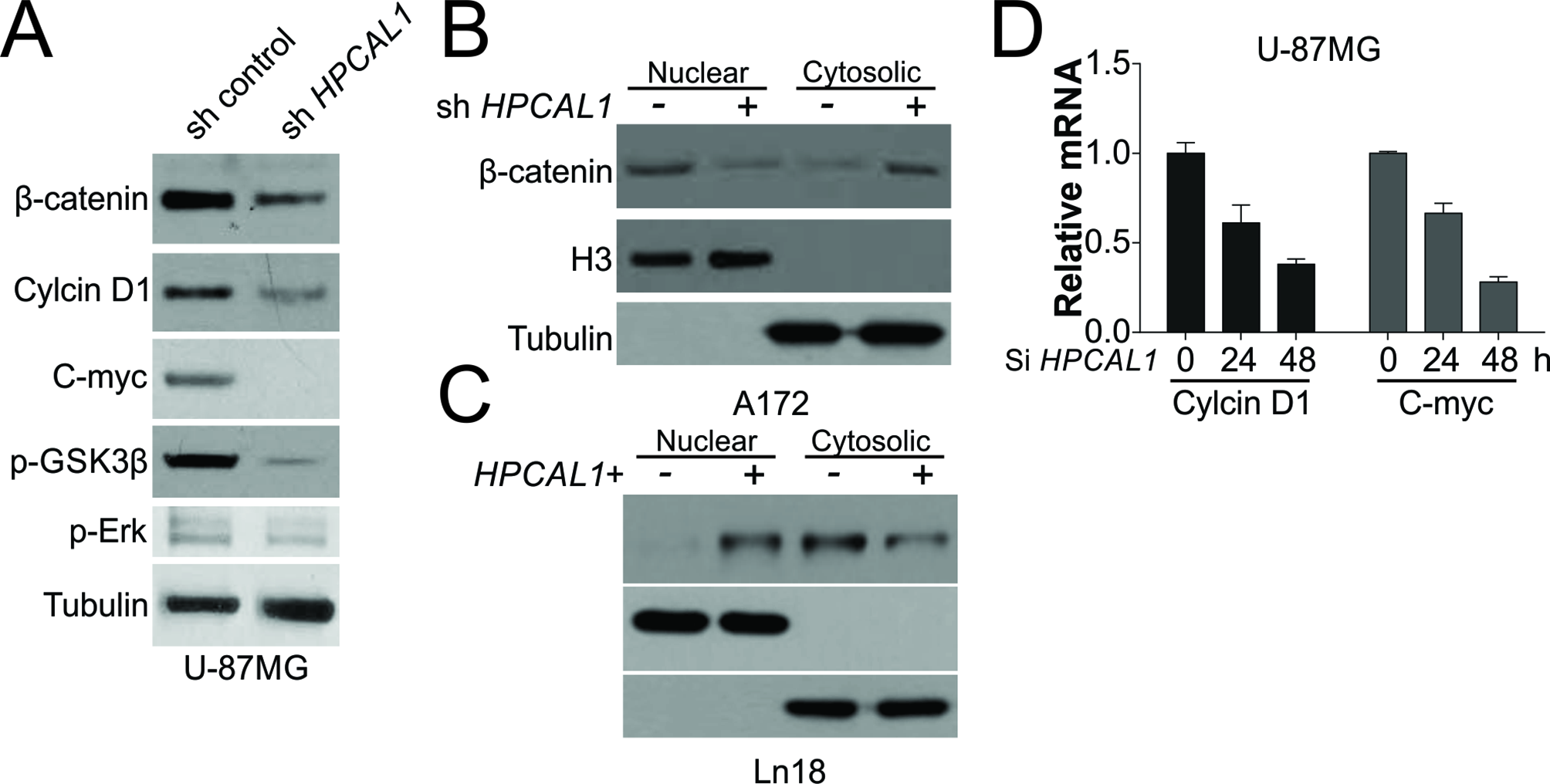

Supplement: Supplementary file 1 [file JCMM-23-3108-s001.tif]

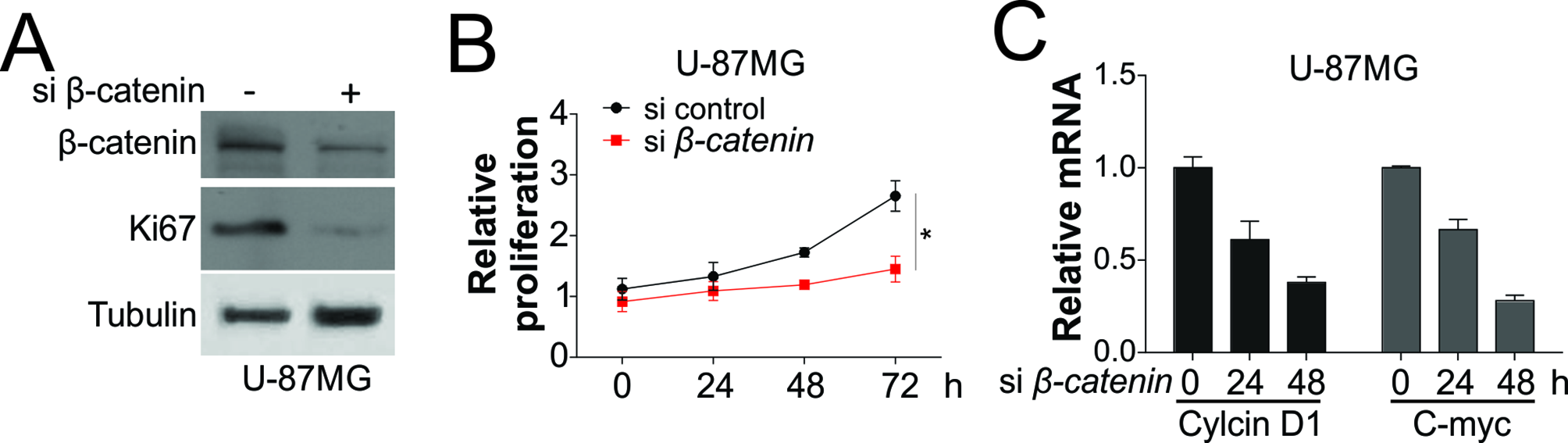

Supplement: Supplementary file 2 [file JCMM-23-3108-s002.tif]

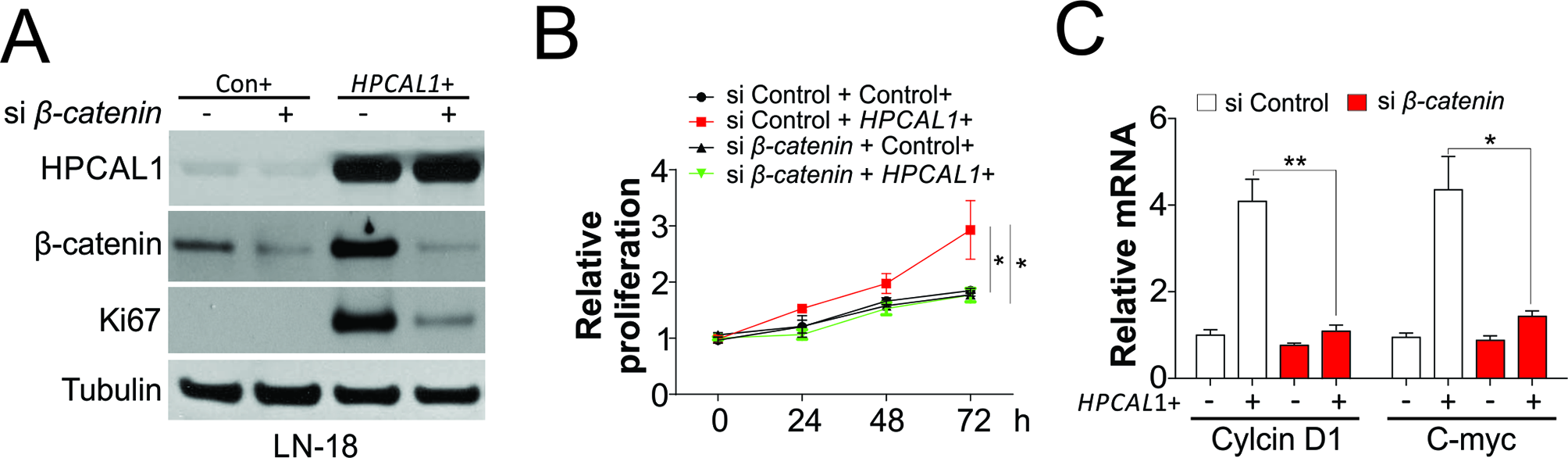

Supplement: Supplementary file 3 [file JCMM-23-3108-s003.tif]

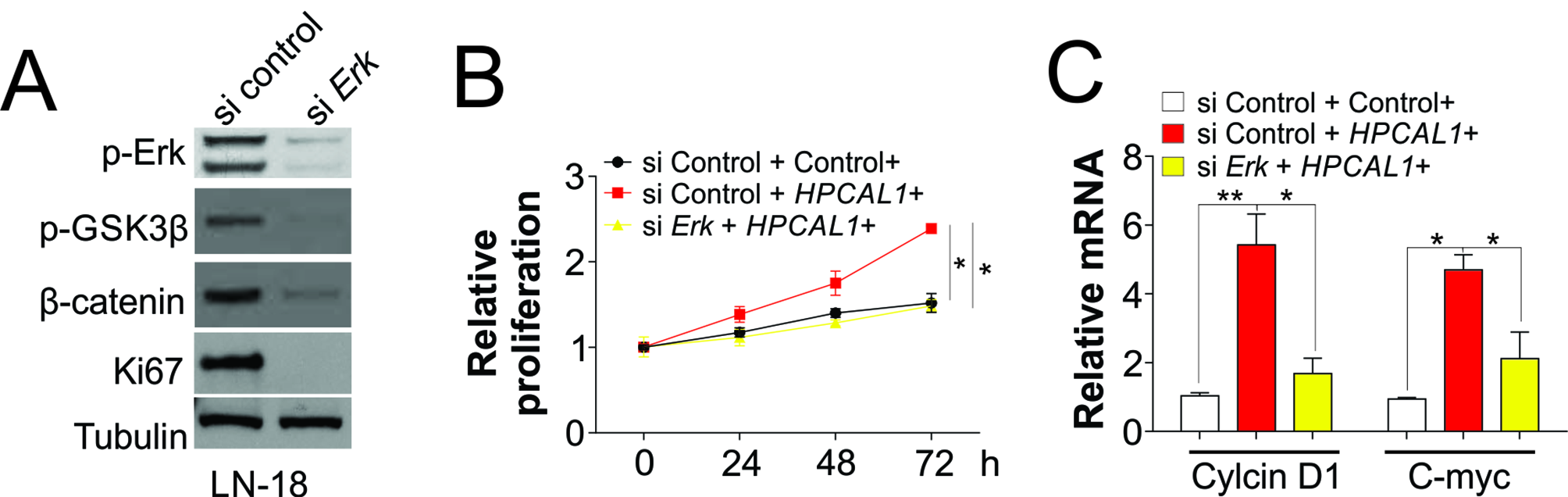

Supplement: Supplementary file 4 [file JCMM-23-3108-s004.tif]
